# A guide for endometrial cancer cell lines functional assays using the measurements of electronic impedance

**DOI:** 10.1007/s10616-017-0149-5

**Published:** 2017-10-07

**Authors:** Joanna Kozak, Paulina Wdowiak, Ryszard Maciejewski, Anna Torres

**Affiliations:** Laboratory of Biostructure, Department of Normal Anatomy, Medical University of Lublin, 20-090 Lublin, Poland

**Keywords:** Endometrial cancer cell lines, Migration, Proliferation, Real-time, Label-free, Cell index

## Abstract

Endometrial cancer cell lines are critical tools to investigate the molecular mechanism of tumorigenesis using the end point cell-based assay such as proliferation, cytotoxicity, apoptosis, anoikis or migration and invasion. The proper assay optimization and performance is essential for physiologically relevant results interpretation. In this study we use label-free real-time cell analysis platform (*xCELLigence*) to optimize growing conditions for proliferation and migration experiments of two types of endometrial cancer cell lines HEC-1-B, HEC-1-A, KLE, and Ishikawa. Profiling of cell lines by cell index measurement in proliferation and migration experiments was performed. Our experimental approach allowed us to monitor particular stage of the cell growth, to see the relation between seeding density and dynamic cell growth as well as to choose the optimal serum concentration as chemoattractant in migration experiment. The highest rate of proliferation was shown for Ishikawa cells. The rapid pace of cellular migration was observed in case of KLE and HEC-1-B cells as compared to weak migratory activity of Ishikawa cells. The cell index that reflects the cell status characterized real-time cytological profile of each analyzed cell line. These cell profiles were crucial for better planning the classical end-point assays used in further research.

## Introduction

Endometrial cancer cell lines derived from two types of tumors are critical tools to study molecular mechanisms underlying particular types of gynecological malignancy. Depending on type of research such properties as cell proliferation, apoptosis, migration or invasion are tested under different experimental conditions. Detailed knowledge about the cell growth kinetics could improve biochemical studies in the direction of developing new agents that modulate pathological processes occurring in cancerous cells.

In this report we profiled the most widely used endometrial cell lines Ishikawa (type I), HEC-1-A (type II), HEC-1-B (type II) and KLE (type II) to provide valuable data such as adhesion characteristic, unique proliferation pattern and migration potency which may be used for optimization of assay performance during experimental design process (Korch et al. 2012). Moreover, unique cell growth patterns presented in this report can be used as a cell line quality control test (Atienza et al. 2005, 2006). Classification of endometrial cancer is based on Bokhman’s classification complemented with detailed genetic, biochemical and histopathological studies. Two types of this carcinoma are now recognized based on differing etiopathological, genetic and clinical characteristics: Type I EC which represents high expression of estrogen and progesterone receptors, low histological differentiation, presenting as an early stage disease. Type II EC represents highly aggressive and invasive cancer with lower expression of estrogen and progesterone receptors than type I, commonly presenting as an advanced stage disease (Bansal et al. 2009; Fong and Meng 2014; Husing et al. 2016).

In our experiments we used the dual plate real-time cell analyzer *xCELLigence* system (RTCA DP) to assess endometrial cell lines proliferation and migration potency under different growth conditions. The RTCA DP Instrument consists of the following main components electronic sensor analyzer and control unit with software. The electronic sensor analyzer contains three cradle pockets each for one E-16 or CIM plate. During the experiment, the electronic sensor analyzer with plates containing the cells is placed inside a cell culture incubator. This label free and operator independent system measures the electronic impedance of sensor microelectrodes incorporated into each well bottom of E-16 plate or CIM plate for proliferation and migration experiments, respectively (Dowling et al. 2014). The electronic impedance value of each well containing the cells is automatically monitored by the *xCELLigence* system for the whole duration of the experiments and depends on the cell attachment to the electrodes. In the absence of cells, electrode impedance is small. In the presence of cells electrode impedance increases. Thus, the more cells are detected by the electrodes, the larger change in electrode impedance occurs (Atienza et al. 2005). The measured electrodes impedance that represent cell status is expressed by a software as a unit-less parameter, called a cell index (CI). In this case CI is a quantitative measure of the cell status as cell attachment to the well bottom, number of cells in the well and cell morphology (Atienza et al. 2006). This useful label-free method allows monitoring cells properties for any set time period.

## Materials and methods

### Cell culture

Endometrial carcinoma cell lines HEC-1-B (ATCC® HTB-113™), HEC-1-A (ATCC® HTB112™) and KLE (ATCC® CRL1622™) were purchased from ATCC (American Type Culture Collection, Manassas, VA, USA) and Ishikawa was purchased from Sigma-Aldrich (St. Louis, MO, USA). HEC-1-B cell line was maintained in MEM (Gibco, Thermo Fisher Scientific, Waltham, MA, USA) supplemented with 10% fetal bovine serum (FBS) (Gibco, Thermo Fisher Scientific, Waltham, MA, USA) and 2% penicillin/streptomycin (PAN-Biotech GmbH, Aidenbach, Germany). HEC-1-A cell line was maintained in Mc Coy’s 5A (Gibco, Thermo Fisher Scientific, Waltham, MA, USA) supplemented with 10% FBS and 2% penicillin/streptomycin. The Ishikawa cell line was maintained in MEM supplemented with 5% FBS and 2% penicillin/streptomycin. KLE was maintained in DMEM (Gibco, Thermo Fisher Scientific, Waltham, MA, USA) supplemented with 10% FBS and 2% penicillin/streptomycin. All cells were grown at 37 °C in 5% CO_2_.

### Subculturing procedure

Cells were harvested using standard trypsinization procedure and counted using trypan blue and Countess device (Invitrogen, Thermo Fisher Scientific, Waltham, MA, USA). For cell proliferation experiments the serial dilution of cells in complete growth medium was performed before adding to E-plate. Cells for migration experiments were resuspended in serum-free medium, counted and seeded at the following density for the HEC-1-B and the Ishikawa cell lines 100,000 cells/well and 50,000cells/well for KLE cell line 100,000 cells/well, 50,000cells/well and 20,000 cells/well in a CIM plate.

### xCELLigence real-time cell proliferation experiment

Proliferation experiments were conducted using *xCELLigence* RTCA DP device (Roche Diagnostics GmbH, ACEA Biosciences, Inc., Penzberg, Germany) which was placed in a humidified incubator at 37 °C in 5% CO_2_. Cell proliferation experiments were carried out using 16-wells (E-16) plates. Microelectrodes for impedance detection during cell attachment, spreading and proliferation were attached at the bottom of each well and had electronic connection with computer software. At the beginning 100 μl complete growth medium was added to each well and water was added to space around the wells to avoid evaporation. Plate was incubated 30 min at room temperature in a laminar chamber. After incubation plate was inserted into device and the background impedance was measured. Next, the HEC-1-B, HEC-1-A and KLE cells were seeded in a range from 1.6 x 10^5^ to 5 x 10^3^ cells/well of E-16 plate in 100µl growth medium per well and Ishikawa cells in a range from 64 x 10^3^ to 4 x 10^3^ cells/well of E-16 plate in 100 μl growth medium per well. Plate was left at 30 min at room temperature in a laminar chamber to allow for cell attachment. Finally the plate was inserted into the device and impedance was automatically monitored and expressed as Cell Index value (CI) by the software. Cell proliferation experiments were run for 72 h for HEC-1-A and HEC-1-B cell lines, 150 h for Ishikawa cell line and 168 h for KLE cell line. CI was monitored every 15 min for the whole experiment duration. Three replications of each cell densities were used in the cell proliferation experiment.

### xCELLigence real-time cell migration experiment

**Fig. 1:**
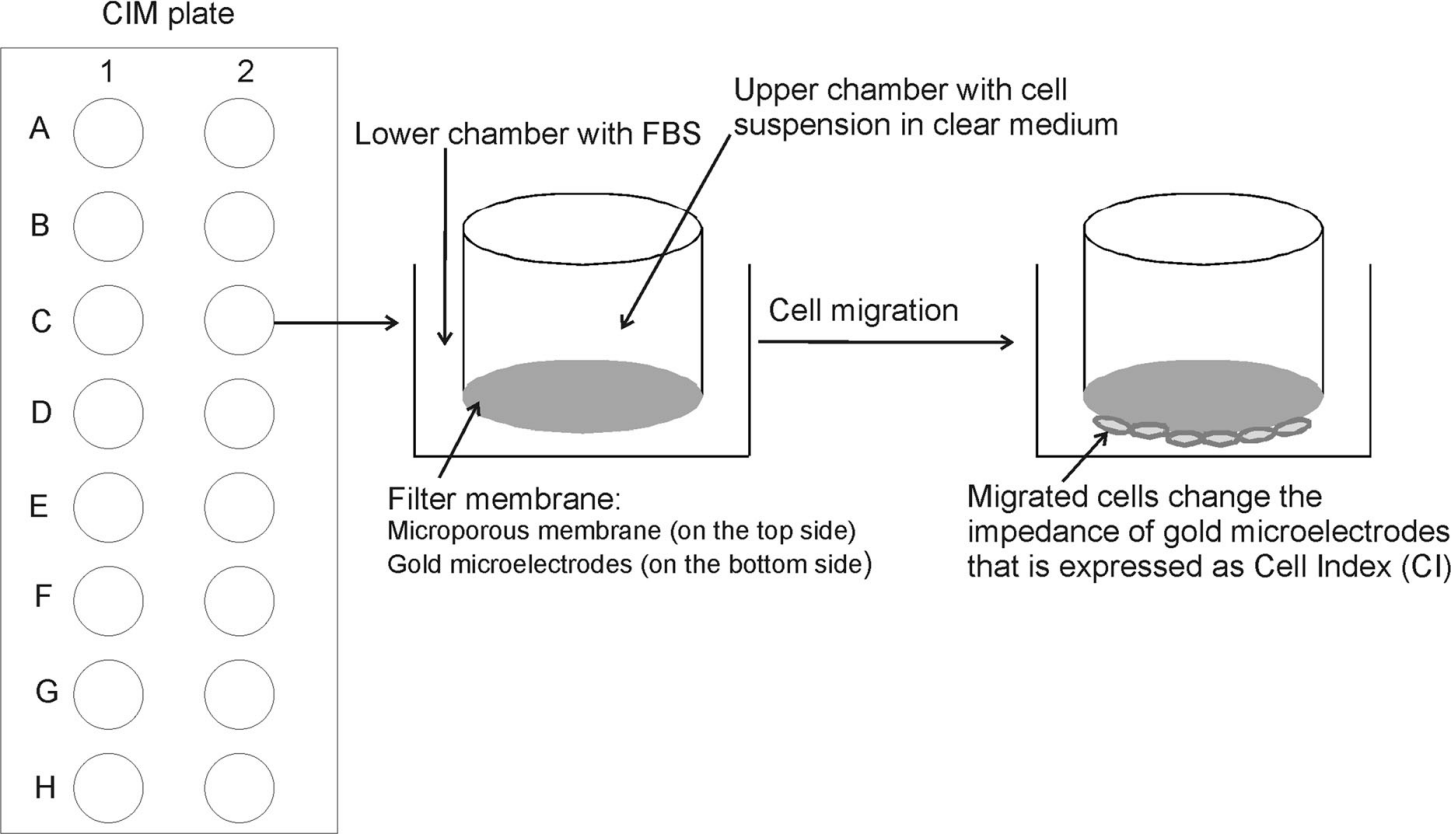
CIM plate scheme. CIM plate containing 16 wells consists of an upper chamber with the cell suspension and a lower chamber (feeding tray) with a chemoattractant. The upper and the lower chambers are separated by a filter membrane consisting of 8 µm-size porous membrane on the top side and microelectrodes on the bottom side. The incorporated microelectrodes detect migrated cells. The serum free cell suspension is seeded into the upper chamber and exposed to chemoattractant that is different FBS concentrations. During the experiment cells migrate toward the lower chamber squeezing through the porous membrane and reaching the incorporated microelectrodes. If cells migrate effectively they generate the electronic impedance signal that is expressed as high CI value

The cell migration experiments were conducted using *xCELLigence* RTCA DP device (Roche Diagnostics GmbH, ACEA Biosciences, Inc) which was placed in a humidified incubator at 37 °C in 5% CO_2_. Presented in Fig. 1 the 16-well CIM plate consisting of an upper chamber and a lower chamber separated by a filter membrane was applied for migration experiments (Fig. 1). Upper and lower chambers fit perfectly each other and form a tight seal. The 8 μm porous membrane permits cell migration toward the bottom side of the upper chamber filter membrane where the microelectrodes are incorporated. The microelectrodes come into contact with migrated cells and generate the electronic impedance signal. The more cells migrate the larger change in electrode impedance occurs that is expressed as high CI value. First, 160 μl complete growth medium with tested FBS concentration 10 or 20% for HEC-1-B and KLE cell lines and 5 or 10 or 20% for Ishikawa cell line was added to each well of the lower chamber then upper chamber was placed onto it in a proper position. Next the 50 μl of clear medium was added to each well of the upper chamber and water was added in the same manner as in proliferation experiments. Plate was incubated for 1 h at room temperature in the laminar chamber. After incubation plate was inserted into the device and the background impedance was measured. Subsequently, the cell suspensions in 100µl per well clear medium were seeded into the upper chamber wells of the CIM plate, as follows: HEC-1-B and Ishikawa cell lines 100,000 cells/well and 50,000 cells/well and KLE cell line 100,000 cells/well, 50,000 cells/well and 20,000 cells/well. Plate was left for 30 min at room temperature in the laminar chamber. Finally, the plate was inserted into the device and impedance was automatically monitored and expressed as CI by the software. Cell migration experiments were run for 18 h and CI was monitored every 15 min. Three replications of each cell density and FBS concentration were performed in the cell migration experiment.

### Data analysis

The growth curves described in proliferation experiments were exported form the RTCA software as mean CI value. The CIs for particular stage of growth curves were read directly from the RTCA software. All data recorded by the RTCA software in the migration experiment were exported to MS Excel for further processing. Raw CI values were selected for respective time points without normalization. The mean and SD were calculated using MS Excel. Comparisons between two independent groups were performed using Student’s *t* test. Statistical significance was determined by *p* value of less than 0.05. Statistical analyses were performed using GraphPad Prism software.

## Results

### Real-time monitoring of cell proliferation

**Fig. 2:**
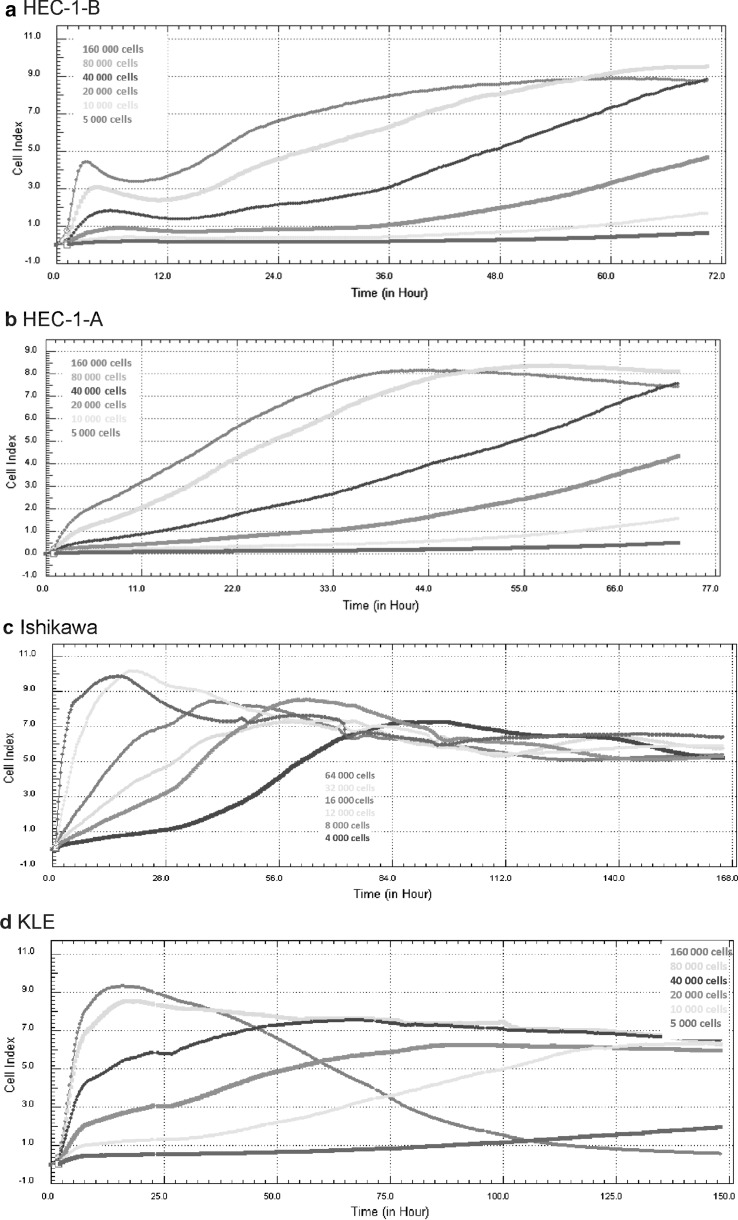
Optimization of endometrial cancer cell lines culture condition using the real-time monitoring *xCELLigence* system. Density and time-dependent growth and proliferation curves at different seeding cell densities are shown. **a** HEC-1-B 5000–160,000 cells/well. **b** HEC-1-A 5000–160,000 cells/well. **c** Ishikawa 4000–64,000 cells/well. **d** KLE 5000–160,000 cells/well

In this study we monitored time-dependent and density-dependent cell proliferation using the RTCA platform of different EC cell lines. The HEC-1-B, HEC-1-A and KLE cells were seeded in a range from 1.6 × 10^5^ to 5 × 10^3^ cells/well of E-16 plate and Ishikawa cells in a range from 64 × 10^3^ to 4 × 10^3^ cells/well of E-16 plate. Cells were automatically monitored for 72, 150 or 168 h and signals were collected every 15 min and expressed as CI value. The monitored time-dependent CI showed distinct growth kinetic profile for each cell line (Fig. 2). This two first stages were the most characteristic of HEC-1-B cells at concentration of 16 × 10^5^, 8 × 10^4^ and 4 × 10^4^ cells/well. For the HEC-1-B line a fast increase of CI in the very first hours after cells seeding was observed and was followed by the CI decrease observed between 3rd and 5th hour of the experiment. The CI recovery indicated the lag phase of HEC-1-B cells. The densities of 16 × 10^5^, 8 × 10^4^ and 4 × 10^4^ cells/well reached the maximum CI at 59, 64 and more than 70 h after cells seeding. The HEC-1-B cell line was characterized by long duration of logarithmic growth phase. The growth curve for 20,000 cells/well had a correct profile but lower maximum CI. Cultures seeded at 10,000 cells/well and 5000 cells/well did not produce any detectable CI read (Fig. 2a).

Lack of CI fluctuation in cell attachment and spreading stages was characteristic for HEC-1-A cells. The growth curve after cell seeding showed a linear adhesion period that was followed by continuous increase of CI for all tested densities. The densities of 16 × 10^5^, 8 × 10^4^ and 4 × 10^4^ cells/well reached the maximum CI at 42, 54 and 70 h after seeding. HEC-1-A cell line was characterized by long duration of logarithmic growth phase. Growth curve for 20,000 cells/well showed a correct profile but had a lower maximum CI. Growth curve for 10,000 cells/well and lower did not produce CI reads (Fig. 2b).

Ishikawa cells were characterized by dramatic and steep increase in the CI in adhesion and spreading stages followed by fast increase of CI in lag phase and exponential growth phase. These data indicated that Ishikawa cells were a fast proliferating cell line that was also corroborated by visual observation (data not shown). Seeding densities of 64,000 and 32,000 cells/well reached the max CI 13 and 19 h after seeding and exhibited a substantial reduction in the CI, which was not suitable for functional assay. Seeding densities of 16,000 and 12,000 cells/well reached the maximum CI 39 and 58 h after seeding which was a relatively slower increase in the CI compared to the seeding densities of 64,000 and 32,000 cells/well. Additionally those two densities exhibited a substantial reduction in the CI after reaching the peak. Growth curves describing densities of 8000 and 4000 cells/well had a correct profile with visible lag phase and exponential growth phase and suitable maximum CI, however both of them were prone to substantial reduction in the CI (Fig. 2c).

KLE cells densities of 160,000 and 80,000 cells/well were characterized by steep increase in the CI in adhesion and spreading stages followed by fast increase of CI in lag phase and exponential growth phase. Furthermore, those two densities reached the max CI after 13 and 16 h, respectively. The seeding density of 160,000 cells/well led to a steep decrease of CI whereas seeding density of 80,000 cells/well led to a small reduction in the CI which indicated a stable plateau phase. Growth curves for 40,000, 20,000 and 10,000 cells/well had correct profiles with optimal period of lag phase and exponential growth phase but lower maximum CI. The seeding density of 5000 cells/well did not produce a CI read at all (Fig. 2d).

### Real-time monitoring of cell migration

#### Optimization experiment of EC cell lines migration condition

In this experiments we investigated how cell seeding densities, serum concentration and cell starvation influences migration of EC cell lines HEC-1-B, KLE and Ishikawa. HEC-1-A was excluded from optimization experiments due to uselessness of this cell line in our other study on microRNA implication in endometrial cancer biology. Therefore we focused on HEC-1-B, Ishikawa and KLE cell lines in migration optimization experiments because we used these results for further experiments. In this study, the optimal cell migration condition was interpreted as the highest CI value reached by cells in particular experimental condition. Cells were monitored using *xCELLigence* CIM plate system with a 8 µm pore size. The rate of migration expressed as CI was monitored over 18 h as cells passed through pores and were detected by the electrodes build into the CIM plate and the mean CI ± SD was calculated at 4, 8, 12 and 18 h.

**Fig. 3:**
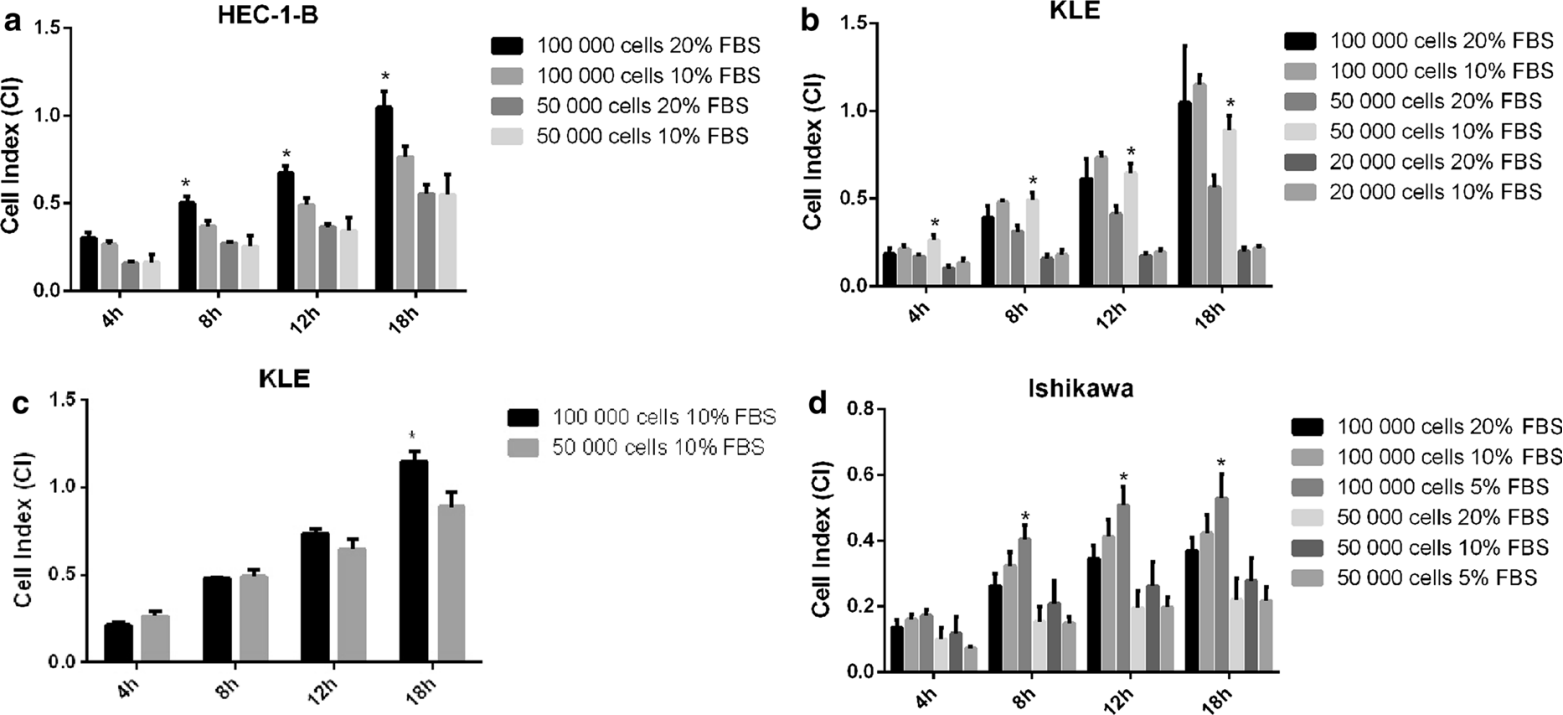
Optimization of endometrial cancer cell lines migration condition using the real-time monitoring *xCELLigence* system. Comparison of the CI between different cell seeding densities migrating towards different FBS concentrations at 4, 8, 12 and 18 h. **a** HEC-1-B cells rate of migration. Seeding density of 100,000 cells/well and 20% of FBS was considered optimal and used for further experiments. *p* values at 8–18 h 20% FBS versus 10% FBS were: 0.0102, 0.006, 0.0123. **b** KLE cells rate of migration. *p* values at 4–18 h 20% FBS versus 10% FBS were: 0.0067, 0.0052, 0.005, 0.0071. **c** KLE cells rate of migration. *p* value at 18 h 100,000 cells/well versus 50,000 cells/well was 0.0118. Seeding density of 100,000 cells/well and 10% of FBS was considered optimal and used for further experiments. **d** Ishikawa cells rate of migration. Seeding density of 100,000 cells/well and 5% of FBS was considered optimal and used for further experiments. *p* values at 8–18 h 20% FBS versus 5% FBS were: 0.0115, 0.016, 0.0294. Data are represented as mean ± SD. *p value < 0.05

Migration of HEC-1-B cells was detected at densities of 100,000 and 50,000 cells/well and at 10 and 20% of FBS. The results indicated the gradual increase in CI with the maximum CI was reached at 18 h of experiment. The cell migration was promoted by 20% FBS concentration comparing to 10% concentration in standard growth medium. The optimal HEC-1-B cells migration conditions, based on CI value, were set as 100,000 cells/well density and 20% concentration of FBS. Little difference in cell migration was observed between 10 and 20% FBS concentration at 50,000 seeding density (Fig. 3a).

Migration of KLE cells was detected at three different seeding densities: 100,000, 50,000 and 20,000 cells/well and two FBS concentrations: 10 and 20%. In the chart on Fig. 3b the statistical significance of different FBS concentration for different cell seeding densities is compared. The results indicated gradual time-resolved increase in CI and significantly higher CI was observed for 10% FBS at 50,000 cell/well seeding density for each time point and at 100,000 cell/well seeding density at 18 h of experiment (Fig. 3b). Based on this observation for further statistical analysis two different cell densities 100,000 and 50,000 cells/well and 10% FBS concentration were chosen. Figure 3c compares the statistical significance of 100,000 and 50,000 cells/well at 10% FBS. This analysis of CI value was used to determine the optimal KLE cells migration condition that was set as 100,000 cells/well and 10% FBS. Cell migration was not promoted by higher FBS concentration. A minor change in CI was recorded at the seeding density of 20,000 cells/well and 20 or 10% FBS concentration (Fig. 3b).

Migration of Ishikawa cells was detected at two different seeding densities 100,000 and 50,000 cells/well and three different FBS concentrations 5, 10 and 20%. The results indicated the gradual increase in CI over experiment time period and significantly higher CI was measured for 5% FBS for each time point at seeding densities 100,000 cells/well. Interestingly, lower seeding densities 50,000 of cells/well were characterized by better migration rate at 10% FBS concentration than at 5 or 20% FBS concentration. The optimal Ishikawa migration conditions, based on CI value, were set as 100,000 cells/well and 5% FBS that corresponded to standard growth medium FBS concentration. Cell migration was not promoted by higher FBS concentration at this particular seeding density. A minor change in CI was recorded at the seeding density of 50,000 cells/well with little or no difference between 20 and 5% FBS concentration (Fig. 3d).

**Fig. 4:**
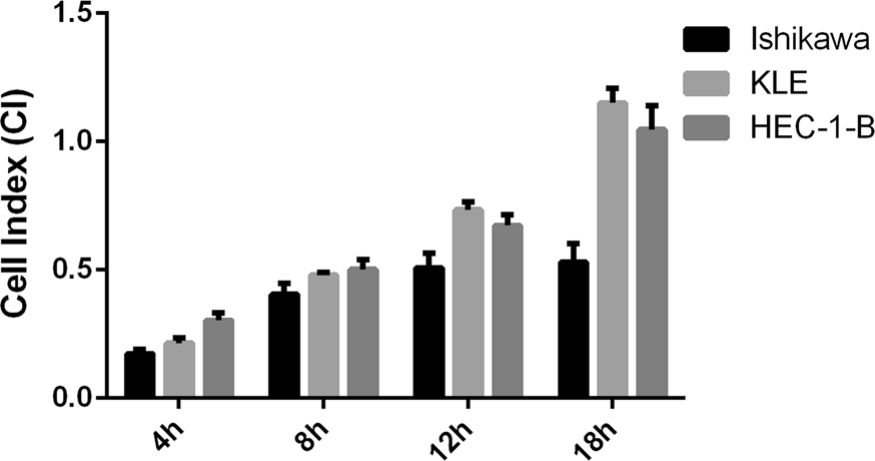
Cells migration rate at optimal condition of experiment. The highest CI value at different time points of migration experiment is compared between endometrial cancer cell lines. The same seeding density of 100,000 cells/well was applied for all cell lines. The FBS concentration was 5% for Ishikawa cells, 10% for KLE cells and 20% for HEC-1-B cells. The rapidest pace of cellular migration among these three cell lines was observed for KLE cells with a maximum CI of 1153 ± 0051 at 18 h of experiment. This is followed by HEC-1-B cells with a maximum of CI 1.04 ± 0.09 at 18 h of experiment and the Ishikawa cells with a maximum of CI 0.52 ± 0.07 at 18 h of experiment. Data are represented as mean ± SD

The optimal migration condition for all tested EC cell lines were determined as follows: 100,000 cells/well seeding density and FBS concentrations of 20% for HEC-1-B, 10% for KLE and 5% for Ishikawa cell line. These conditions were used to compare and determine which cell line has the highest migratory potency. In this study, the migratory potency was correlated to CI value reached by the cells and was compared as such. As shown in Fig. 4 HEC-1-B and KLE cells showed higher migratory potential than Ishikawa cells for each analyzed time point. HEC-1-B cells reached higher CI at 4 and 8 h than KLE cells. KLE cells increased migration rate at 12 and 18 h and reached higher CI value than HEC-1-B cells at the same time point (Fig. 4). These data indicate that KLE cell migration is the highest compared with that achieved by HEC-1-B and Ishikawa cells.

#### Cell migration in response to 4 h serum starvation

To test whether cell serum starvation could promote or inhibit migratory potency of the studied cell lines we employed the results obtained during optimization *experiment of EC cell lines migration condition*. Therefore 100,000 cells/well seeding density was chosen for all tested EC cell lines. Moreover, the FBS concentrations were chosen as follows: 20% for HEC-1-B, 10% for KLE and 5% for Ishikawa cell line (Fig. 4). Four hours prior to conducting the migration experiments all EC cell lines were deprived of serum. The migratory potential of serum–starved cells was determined by CI monitoring over 18 h and the mean CI ± SD was calculated at 4, 8, 12 and 18 h.

**Fig. 5:**
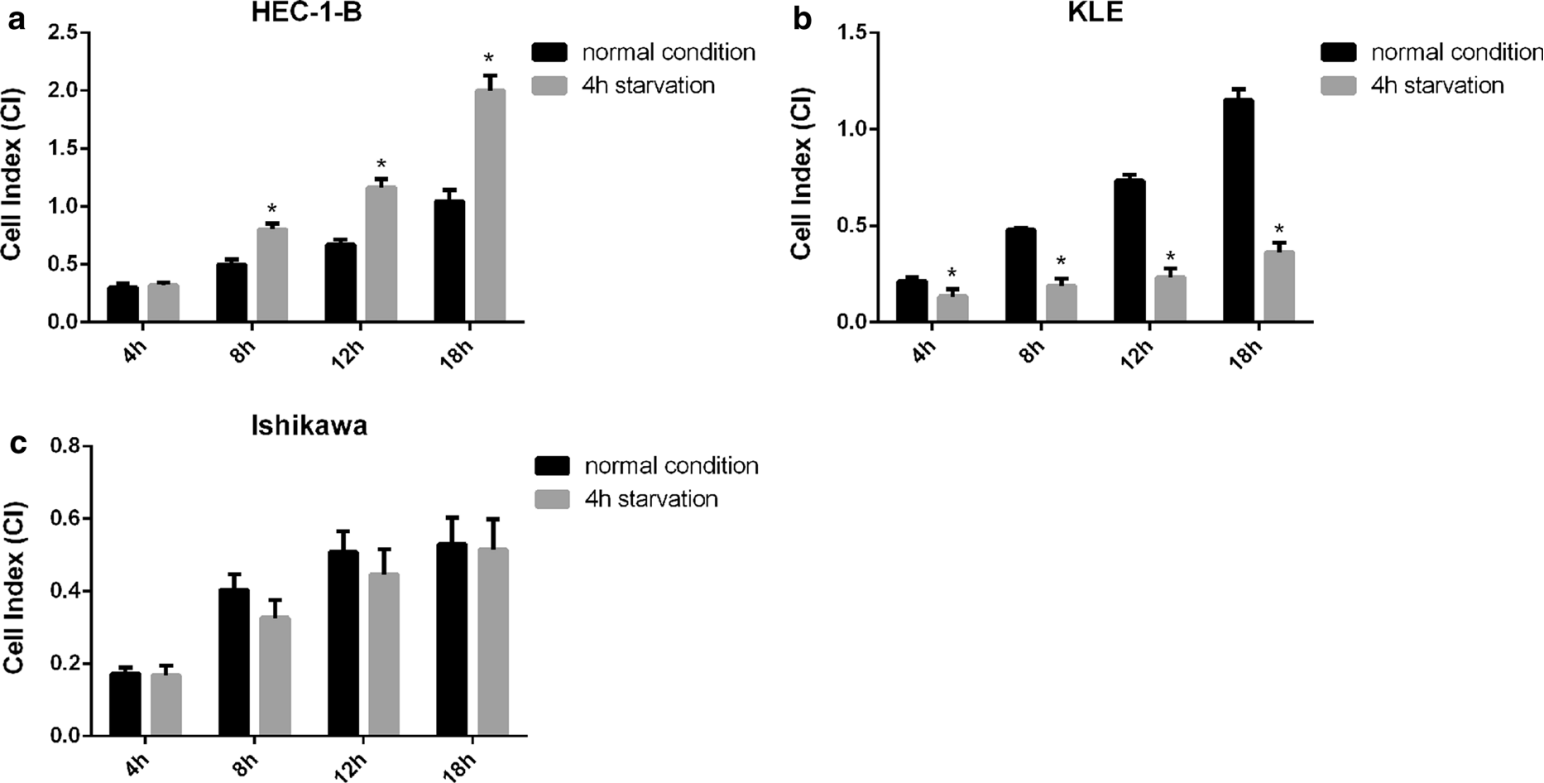
Effect of the serum starvation on the migration rate of endometrial cancer cell lines. Comparison of the cells motility expressed as CI between the different growth conditions. The normal condition indicates cells overnight growth in complete medium before migration experiments and 4 h starvation indicates 4 h cells growth in medium without serum before migration experiments. CI is compared at 4, 8, 12 and 18 h of experiment. **a** HEC-1-B cells rate of migration is significantly enhanced at 8, 12 and 18 h of experiment in 4 h starvation condition than under normal conditions. *p* values at 8–18 h were: 0.0003, 0.0001, 0.0001. **b** KLE cells rate of migration is negatively affected by serum deprivation at each experimental time point. Significant differences between normal condition and 4 h starvation are observed at 4, 8, 12 and 18 h. *p* values at 4–18 h were: 0.0234, < 0.0001, < 0.0001, < 0.0001. **c** Ishikawa cells rate of migration is slightly inhibited by serum deprivation at 8 and 12 h of experiment. No significant differences are observed between normal condition and 4 h starvation over the course of migration experiment. Data are represented as mean ± SD. *p value < 0.05

Serum starved HEC-1-B cells showed enhanced migration capabilities. It was found that the HEC-1-B cells exhibited increased CI under starvation experimental condition as compared to experiment condition without starvation at each analyzed time point (Fig. 5a).

Serum starvation had a negative impact on motility of KLE cells as compared to normal migration condition. A dramatic decrease in migration capabilities was observed at each analyzed time point (Fig. 5b).

It was found that Ishikawa cells motility was not significantly modulated after serum starvation. However, the CI reduction was observed at 8 and 12 h of experiment as compared to normal migration condition. No significant difference of CI at 4 and 18 h of experiment was observed in both experimental condition (Fig. 5c).

Collectively, our results revealed that HEC-1-B and KLE cells showed higher migratory potential than Ishikawa cells (Fig. 4). Moreover, serum deprivation enhanced HEC-1-B cells migration capabilities, diminished KLE cells migration capabilities and had no or little impact on Ishikawa cells migration capabilities.

## Discussion

The endometrial cancer cell lines used in our study are widely accepted models to investigate the molecular mechanisms of female reproductive tract tumors. The Ishikawa cell line is an excellent model for type I endometrial cancer and HEC-1-A, HEC-1-B and KLE cell lines correspond to type II endometrial cancer (Albitar et al. 2007; Korch et al. 2012). The well-differentiated Ishikawa cell line was originally established from an endometrial adenocarcinoma from a 39-year-old Japanese patient. The cells are estrogens and progesterone receptor positive (Nishida 2002). The proliferation rate of Ishikawa cells, expressed as doubling time, increases with the number of passages (Nishida 2002). The Ishikawa cell line represents the histological grade 1 (G1) endometrial tumor (Diaz-Valdivia et al. 2015). The HEC-1-A and HEC-1-B were established from an endometrial adenocarcinoma from a 71-year-old patient (Lelle et al. 1993). HEC-1-B cell line is a substrain of HEC-1-A. Both cell lines are histologically consistent with grade 2 (G2) endometrial tumor (Lelle et al. 1993). The KLE cell line was established from 65-year-old women in advanced disease. The cells were obtained from a metastasis to the colon. The KLE cell line is histological classified as poorly differentiated grade 3 (G3) endometrial tumor (Lelle et al. 1993). The comprehensive molecular characteristic of the endometrial cancer cell lines faithfully replicate the histological and morphological features of type I and II endometrial cancer. Furthermore, these two types of endometrial cancer represented by Ishikawa, HEC-1-A, HEC-1-B and KLE cell lines show a strong differences in motility and proliferation rate that was observed in our study and is in agreement with other researchers’ results (Bansal et al. 2009; Goto et al. 2008; Lu et al. 2015; Winship et al. 2016). Modulation of gene expression pattern, cellular signaling pathways changes and receptors activity induced by plethora of therapeutics tested in multiple studies, disclosed various morphological changes in endometrial cancer cells structure, as well alterations in proliferation rate, survival, migration and invasion (Bansal et al. 2009; Diaz-Valdivia et al. 2015; Lelle et al. 1993). The precise measurement of changes in cellular behavior is the most critical aspect of functional assays. Only verifiable and replicable study results make the promising step toward new therapeutics development. The advanced electronic devices such as the one utilized in the presented study, come up to high expectations in the field of optimization experimental conditions with endometrial cancer cell lines. The current study is the first to present the detailed characteristic of proliferation and migration experimental conditions of type I and II endometrial cancer represented by Ishikawa and HEC-1-A, HEC-1-B, KLE cell lines.

Our experiments revealed that HEC-1-B cells entered into logarithmic growth phase approximately 12 h after seeding for all tested densities which enabled enough time for cell growth modulation prior to reaching maximum CI value. These results are in accordance with the results presented by Yan et al. (2014) and also by Ni et al. (2015). Our present results thus confirm the importance of considering the time between cell seeding and positive or negative cells growth modulation. As observed in our study, the most appropriate rate of CI increase for HEC-1-A cell line gave good opportunity to monitor tested compound interference with the cells in cytotoxicity or viability experiments. The same optimal HEC-1-A cells number which was observed in our study was used by Wong et al. (2008), who determined the effect of dichloroacatate on the viability of endometrial cancer cells. In our proliferation experiments we chose different cell seeding concentration ranges for Ishikawa cell line than for HEC-1-A, HEC-1-B and KLE. Our choice was based on visual cell culture observation that indicated the Ishikawa cells as the fastest proliferating cell line. Moreover, we observed that proliferation rate of Ishikawa cells increased with the number of passages what is consistent with Nishida et al. (2002) observation. This phenomenon could be connected with Ishikawa cell line classification as type I endometrial tumor (Albitar et al. 2007; Korch et al. 2012). A similar observation of increasing proliferation rate with increasing numbers of passages was not observed for HEC-1-A, HEC-1-B and KLE. Avoiding the plate walls overloading with cells suspension we decided to start proliferation experiments with lower seeding density for Ishikawa cell line than for the rest of tested EC cell lines. The low seeding density gave us the opportunity to observe the adhesion and spreading stages of the Ishikawa cell line. Those observations are supported by the results presented by other researchers. Schaefer et al. (2010) used similar cell concentration to determine the cytotoxic effect of cadmium chloride on Ishikawa cells by real-time *xCELLigence* cell impedance analysis. The cell plating concentration, time for cell attachment and spreading as well as compound incubation time presented by Winship et al. (2016), also supported our observation about Ishikawa cells growth characteristic. The optimum seeding concentration for KLE cell line in our experiment was established in range of 40,000–20,000 cells/well. At this cell density the gradual increase of CI was suitable for any cells growth manipulation and CI changes were monitored easily. This optimum seeding concentration for KLE cell line could be used during widely acceptable end-point assays and is supported by some another authors’ experiments (Wong et al. 2008).

The *xCELLigence* system was used in our migratory study to measure the impedance change across the CIM plate as cells squeezed through the pores. This electronic method facilitated more accurate detection of the migration process than traditional assays of staining and counting migrated cells. In order to consider the positive migration signal, the CI value should be greater than or equal to 0.1. In this purpose we applied the 20,000, 50,000 and 100,000 cells/well cell seeding densities for all examined cell lines. Analysis of the CI for 20,000 cells/well seeding density for HEC-1-B and Ishikawa cell lines, revealed CI values at a level of 0.008 and 0.002, respectively. For these particular cell lines our results were not satisfactory therefore we decided not to present them in manuscript. The 20,000 cells/well seeding density for KLE cell line gave us a CI value at a level of 0.2 and we considered them for publication. We stimulated cells of the studied EC lines to migrate toward chamber with FBS gradient selected according to two criteria. First, the FBS concentration as a chemoattractant was the same as the complete growth medium FBS concentration for the particular cell line. Second, the FBS concentration as a chemoattractant was two times higher than in complete growth medium to check if it is a sufficiently strong chemoattractant concentration to induce the directional migration of the cells through micropores of the CIM plate. The complete growth media for the HEC-1-B and KLE cell lines are supplemented with 10% FBS therefore based on our criteria we have chosen 10 and 20% FBS concentration for migration experiments. The growth medium of the Ishikawa cell line is supplemented with 5% FBS in our laboratory. However, we found that Saegusa et al. (2008) and Park et al. (2012) cultivate the Ishikawa cells in medium supplemented with 10% FBS therefore in our experiments we applied 5, 10 and 20% FBS. Our results showed that among the tested cell lines HEC-1-B expressed the highest migratory potency at 20% FBS as chemoattractant. Moreover, our migration study indicated that the serum deprivation improved migration rate of HEC-1-B cells. These results are in agreement with observations presented by Acconcia et al. (2006), and are in line with aggressive characteristic of type II EC represented by HEC-1-B cell line (Bansal et al. 2009). The optimal FBS concentration found in our migration experiments was 5% and the 10% for Ishikawa and KLE, respectively, which is supported by other authors’ results (De Barros et al. 2016; Xiong et al. 2016). We established that an increase in the FBS concentration did not augment migration capabilities of Ishikawa and KLE cells. Furthermore, we found that the serum deprivation of KLE cells inhibited cells migration. We speculate that such phenomenon could be attributed to numerous kinds of growth factors, cytokines and hormones present in the serum which contribute to the activation of different signal pathways related to survival, proliferation or migration of the cells. On the basis of findings presented by Acconcia et al. (2006), Hou et al. (2014) and Leblanc et al. (2007), we speculated that inhibition of KLE cell migration could be mediated by estrogen receptors beta (ERβ). It is therefore possible that response to serum deprivation but not serum excess could be cell line specific and mediated by estrogen receptors involved in signaling pathways that play roles in cell motility.

Subsequent observations of Ishikawa cells propagated in the serum deprivation experimental conditions revealed that the migration pattern did not appear to be different from the non-starved cells. Similar weak migratory potency of Ishikawa cells was observed by Winship et al. (2016). The authors suggested that such phenomena might have been due the origin of Ishikawa cells from G1 endometrial cancer, which was characterized as the tumor with low migratory and invasive properties (Winship et al. 2016).

In conclusion, in the present study we used the *xCELLigence* system to indicate the optimal seeding density for proliferation, cytotoxicity or viability studies and to optimize cell density and FBS concentration for migration experiments. The presented results can be applied as valuable predictors for planning experiments with classical end-point proliferation, cytotoxicity or viability and migration assays on EC cell lines and making the result analysis more physiologically relevant. Moreover, the presented results can be applied as valuable predictors for planning expensive and time-consuming in vivo experiments. An important finding of our research was the fact, that each EC cell line presented the unique “kinetic profile” with the real-time monitoring system for proliferation and migration experiments. The precise observations of cells behavior acquired by the microelectrode sensors on E-16 and CIM plates allowed for more physiologically relevant data analysis experiments.
